# Uncovering antibiotic resistance: extended-spectrum beta-lactamase-producing *Pseudomonas aeruginosa* from dipteran flies in residential dumping and livestock environments

**DOI:** 10.3389/fmicb.2025.1586811

**Published:** 2025-06-04

**Authors:** Lara de Wet, Itumeleng Matle, Oriel Thekisoe, Kgaugelo E. Lekota, Tsepo Ramatla

**Affiliations:** ^1^Unit for Environmental Sciences and Management, North-West University, Potchefstroom, South Africa; ^2^Bacteriology Division, Onderstepoort Veterinary Research, Agricultural Research Council, Onderstepoort, South Africa; ^3^Department of Life Sciences, Centre for Applied Food Safety and Biotechnology, Central University of Technology, Bloemfontein, South Africa

**Keywords:** Diptera flies, *Pseudomonas aeruginosa*, antibiotic resistance, virulence genes, WGS

## Abstract

**Introduction:**

*Pseudomonas aeruginosa* poses challenges in clinical and environmental contexts due to its capacity to colonize natural ecosystems and antibiotic resistance. This study characterized *P. aeruginosa* harboured by Diptera flies collected from illegal residential dumping sites and livestock (cattle, sheep, and goats) kraals in Potchefstroom, South Africa.

**Methods:**

The *P. aeruginosa* isolates were characterized using classical microbiological tests and species-specific *gyrase B* gene PCR assay. Antibiotic resistance (AR) was assessed on the isolates using disc diffusion assay (DDA). Additionally, PCR screened six virulence genes (*exoS*, *plcN*, *plcH*, *toxA*, *lasB*, and *algD*) among the isolates. Whole genome sequencing (WGS) was employed to confirm the identity and determine antibiotic resistance genes (ARGs) on selected isolates.

**Results:**

Culture-based and molecular assays showed that *P. aeruginosa* is prevalent in Diptera flies (*Hemipyrellia* spp., *Synthesiomya* spp., *Chrysomya* spp., *Sarchophagidae* spp., and *Tabanus* spp.) from livestock kraals (75%; *n* = 36/48) and dumping sites (48%; *n* = 23/48). The most detected virulent gene among the isolates was *exoS* (96.6%), followed by *plcN* and *algD* genes (83.1%), *lasB* (81.4%), *toxA* (76.3%), and *plcH* (47.5%). All *P. aeruginosa* isolates were resistant to metronidazole, sulphamethoxazole, cefazolin and amoxicillin based on DDA. The sulfonamide resistance *sulI* gene (88.1%) was the most detected ARG from the *P. aeruginosa* isolates, followed by *acc(3)-IV* (80.6%) coding for aminoglycoside. WGS revealed that *P. aeruginosa* isolates belong to the sequence type (ST3808), which is multidrug-resistant and contains ARGs for fosfomycin (*fosA*), ampicillin (*bla*_OXA-50_), chloramphenicol (*catB7*), beta-lactamase (*bla*_PAO_), and aminoglycoside (*aph(3’)-IIb*).

**Discussion:**

This study isolated ESBL-producing *P. aeruginosa* from various Diptera fly species collected from livestock kraals and residential dumping sites. This bacterium is important to “One Health” due to its multidrug resistance character and zoonotic nature. As a result, it requires consolidated control and management policies from the environmental, veterinary, and human health sectors.

## 1 Introduction

Diptera flies are among the most diverse groups of organisms that consist of 128 families and 124,000 species and serve as biological or mechanical vectors that carry and transmit multiple pathogenic bacterial species (Skevington and Dang, 2002; Taioe et al., 2017). Among the present bacterial species in Diptera flies is *Pseudomonas aeruginosa*, which can spread to other ecosystems, such as habitats for animals, humans, and the environment (Pang et al., 2019). It is a versatile, opportunistic bacterium that infrequently affects healthy individuals and is commonly associated with ventilator-associated pneumonia and nosocomial infections (Pang et al., 2019). Due to their mobility, eating, and excretory activities, Diptera flies can spread *P. aeruginosa* throughout settings high in organic waste (Boiocchi et al., 2019). This enhances the bacteria’s capacity to colonize various ecological niches, such as the surfaces of plants, water, and soil. The *P. aeruginosa* can be a major microbial participant in habitats where Diptera flies contribute to decomposition, helping to break down organic materials and affecting nutrient cycling processes (Pang et al., 2019).

It is well recognized that *P. aeruginosa* possesses a wide range of virulence factors, which enhance its pathogenicity and capacity to cause infections. Elastases (*lasA* and *lasB*), which break down elastin and other host proteins and cause tissue damage and invasion, are among the virulence factors (Faraji et al., 2016; Cathcart et al., 2011). Exotoxins also prevent protein synthesis in host cells by ADP-ribosylation of elongation factor-2, which results in cell death. In addition, other virulence factors include the Type VI Secretion System (T6SS), which influences bacterial competition and virulence, and the Type III Secretion System (T3SS), which tampers with cellular functions and immune responses. The coordination of various virulence factors’ expression, including biofilm formation and toxin production, is facilitated by the quorum sensing systems (*Rhl* and *Las*) (Deep et al., 2011).

*Pseudomonas aeruginosa* is known to pose a serious risk to both human and animal health due to its high level of intrinsic resistance to several different classes of antibiotics (Lister et al., 2009; Santajit and Indrawattana, 2016). Carbapenem-resistant *P. aeruginosa* produces extended-spectrum beta-lactamases (ESBLs) and metallo-beta-lactamases (MBLs) enzymes, causing resistance to various beta-lactam antibiotics (Paterson and Bonomo, 2005; Neyestanaki et al., 2014). Beta-lactams’ binding affinity may be decreased by mutations in Penicillin-Binding Proteins (PBPs) (Pandey and Cascella, 2021). Additionally, aminoglycosides are modified by enzymes that change them, making them ineffective. The resistance of *P. aeruginosa* to fluoroquinolones, tetracycline, and chloramphenicol, as well as the poor permeability of the outer membrane, are highly influenced by efflux pumps (Li et al., 1994).

Studies examining *P. aeruginosa* in Diptera flies are scarce, particularly in livestock and waste environmental settings. It is essential to comprehend these relationships and *P. aeruginosa*’s unique function in order to comprehend the traits of antibiotic resistance in this organism. Therefore, this study investigated the occurrence of *P. aeruginosa* and antimicrobial resistance patterns from Diptera flies collected in Ikageng township illegal residential dumping sites and livestock kraals in Matlwang village of Potchefstroom City in the North-West province of South Africa.

## 2 Materials and methods

### 2.1 Collection of Diptera flies

**Figure 1:**
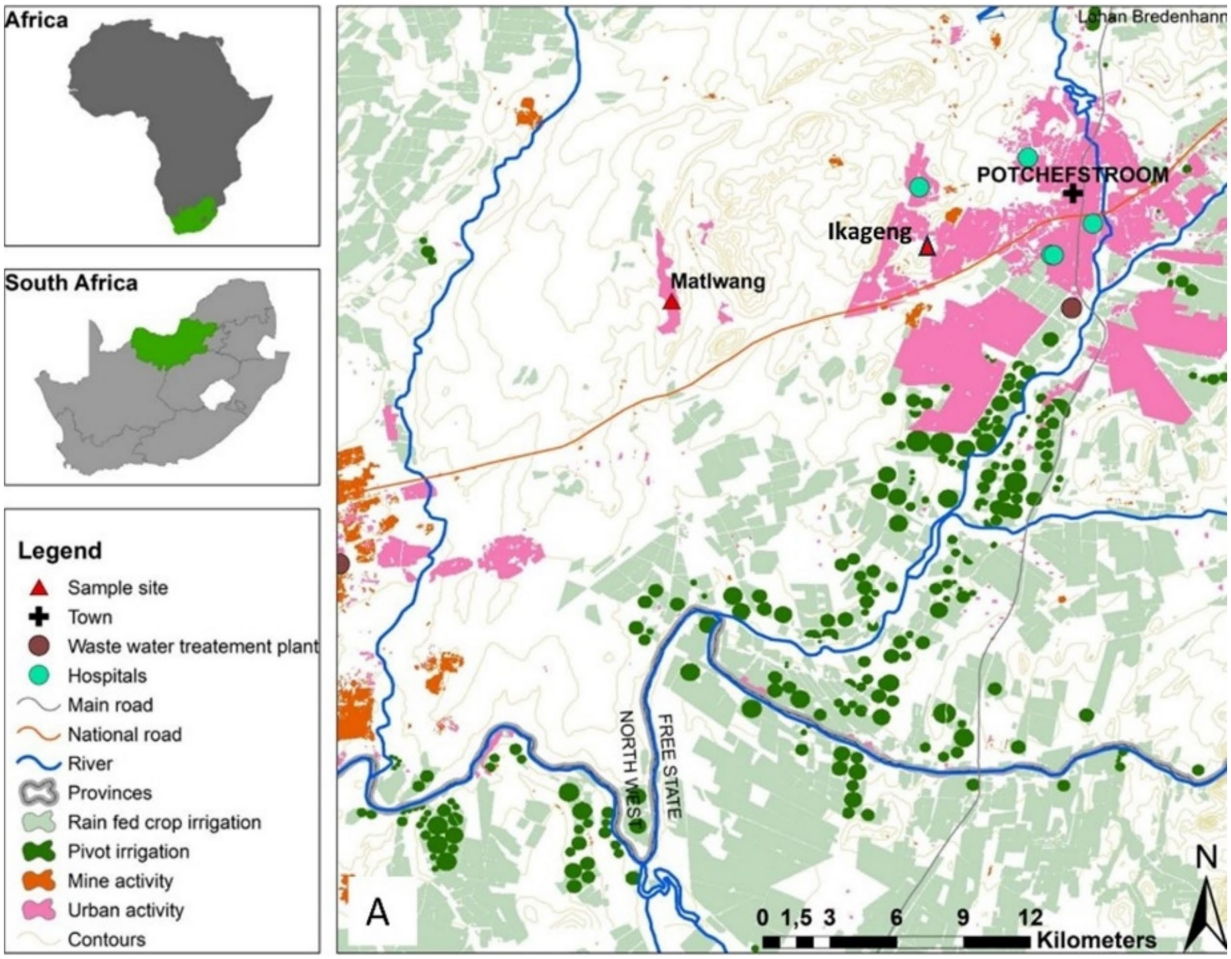
Map of the North West province in South Africa indicating Ikageng residential dumping site and Matlwang community area.

Diptera flies were collected from the illegal residential dumping site at Ikageng township (Coordinates: 26.7257° S; 27.0500° E) and from the Matlwang village livestock kraals (Coordinates: 26.4451° S; 26.5552° E) of Potchefstroom town in the North-West province of South Africa (Figure 1). Redtop disposable fly catcher traps were deployed for 14 days, and the flies were harvested every 2 days. Fly traps were set at two Ikageng illegal residential dumping sites which are 120 m from each other, while fly traps (~60 m from each other) were set in three livestock kraals in Matlwang village.

### 2.2 Identification of Diptera flies using microscopy

Diptera flies were separated according to morpho-species identification as described and then put in a 9:1 ratio of phosphate buffered saline (PBS) to glycerol. The Nikon SMZ1500 stereoscopic zoom microscope was utilized to identify the Diptera flies based on their size, shape, and color.

### 2.3 DNA extraction from Diptera flies

DNA was extracted from fly specimens using the DNeasy^®^ Blood and Tissue kit according to the manufacturer’s instructions (QIAGEN, Germany). The quality and quantity of the extracted DNA was analyzed using spectrophotometry with a NanoDrop ND-100 system (NanoDrop Technologies, Wilmington, United States). The extracted DNA was stored at −20°C until used for PCR analysis.

### 2.4 Genetic identification of the flies

The *Cytochrome Oxidase 1* (*CO1*) gene PCR assay was conducted for the identification of fly species using the following primers: LCO1: 5′-GGTCAACAAATCATAAAGATATTGG-3′ and HCO1: 5′-TAAACTTCAGGGTGACCAAAAATCA-3′. Each PCR reaction consisted of a total reaction of 25 μL consisting of 12.5 μL OneTaq^®^ Quick-Load 2X Master Mix with Standard Buffer (0.4 mM dATP, 0.4 mM dCTP, 0.4 mM dGTP, 0.4 mMdTTP, 4 mM MgCl2, and loading buffer) (New England BioLabs, United States), 8.5 μL of nuclease-free water, 2 μL (5 ng/μl) of the template DNA, and 1 μL (10 μM) of each oligonucleotide primer. The PCR mixture was incubated on a ProFlex PCR system (Applied Biosystems, Singapore) with the following conditions: Initial denaturation at 94°C for 2 min, then 35 cycles of 94°C for 30 s, 47°C for 30 s and 72°C for 60 s, and final elongation at 72°C for 7 min (Monyama et al., 2023). Thereafter, PCR amplicons were sequenced with the BigDye Terminator sequencing kit v3.1 (Applied Biosystems) on the SeqStudio genetic analyzer at UESM sequencing facility of North-West University.

The sequenced gene were edited using FinchTV (Treves, 2010). The forward and reverse strands were linked together using BioEdit (Tippmann, 2004). The fly *CO1* gene sequences were subjected to nucleotide Basic Local Alignment Search Tool (BLASTn) for confirmation of identical sequences available on the GenBank of the National Centre for Biotechnology Information (NCBI). Phylogenetic trees were created using maximum likelihood on Molecular Evolutionary Genetics Analysis (MEGAX) software (Kumar et al., 2018). A bootstrap value of 1,000 was employed to guarantee the tree’s confidence.

### 2.5 Culture isolation and biochemical characterization of *Pseudomonas aeruginosa*

A total of 96 pooled representative samples (5 flies per pool), comprising 45 from dumping sites and 51 from livestock kraals, were used in this study. These were randomly selected from the total collected fly population to ensure manageable yet statistically representative processing for downstream microbial analysis. Only flies morphologically identified as commonly present at both sites were included to ensure ecological comparability and minimize species-specific bias. Unknown or site-exclusive fly species were excluded to maintain consistency in species-related pathogen carriage assessment. To reduce surface contamination, the exterior surface of the flies was disinfected with 70% ethanol for 1 h. The flies were then crushed in phosphate buffer solution (PBS), followed by a 20 s vortexing. Thereafter, a volume of 1 mL of the PBS-fly homogenate was placed in 9 mL buffered peptone water (BPW) and incubated for 24 h with agitation at 37°C. A spread plate method was utilized where 100 μL of the BPW were transferred onto 90 mm agar plates of Cetrimide agar (Biolab) (Denissen et al., 2024). The spread plate method was carried out in duplicate to ensure that the results were harmonized. All the inoculated plates were incubated at 37°C for 24 h for culture isolation. The colonies grown were examined for morphology and pigment production and for gram-staining. Oxidation-Fermentation Test: This test was performed on presumptive *Pseudomonas* species to determine their oxidative reaction. Specifically, among the same samples of oxidase (+), catalase (+), and motility (+) activities (Nepali et al., 2018; Tohya et al., 2022), colonies were studied according to Abdalhamed et al. (2016) to confirm the *Pseudomonas* species. The nutrient agar (NA) (Biolab) plates were incubated for 24 h at 37°C to obtain pure cultures for molecular identification. *Pseudomonas aeruginosa* ATCC 27853 was used a positive control.

### 2.6 Genomic DNA extraction from *Pseudomonas aeruginosa*

DNA was extracted from overnight incubated cultures of *P. aeruginosa* using DNA Mini Kit (Qiagen, Germany) according to the manufacturer’s instructions. DNA quantification was conducted using the NanoDrop ND-100 system (NanoDrop Technologies, Wilmington, United States).

### 2.7 Identification of *Pseudomonas aeruginosa* using *gyraseB* gene PCR

Conventional PCR was conducted to identify *Pseudomonas* spp. isolated from Diptera flies targeting the *P. aeruginosa gyraseB* gene using the following primers: Forward: CCT GAC CAT CCG TCG CCA CAA, and Reverse: CGC AGC AGG ATG CCG ACG CC (Ahmadi et al., 2024). Each PCR reaction was conducted with a total mixture of 25 μL consisting of 12.5 μL OneTaq^®^ Quick-Load 2X Master Mix with Standard Buffer (0.4 mM dATP, 0.4 mM dCTP, 0.4 mM dGTP, 0.4 mMdTTP, 4 mM MgCl2, and loading buffer) (New England BioLabs), 8.5 μL of nuclease-free water, 2 μL of the template DNA, and 1 μL of each oligonucleotide primer. PCR conditions were as follows: 96°C for 4 min, 35 cycles of 94°C for 30 s, 57°C for 30 s, 72°C for 1 min, and one step of final elongation at 72°C for 10 min with an infinite hold at 4°C. The amplified PCR products were electrophoresed on 1.5% agarose gel, stained with Ethidium Bromide (EtBr) and visualized under UV light.

### 2.8 The bacterial *16S rRNA* gene PCR and sequencing

The *16S rRNA* gene PCR assay was conducted using universal primer pair namely, 27F-GAGTTTGATCCTGGCTCAG and 1492R -GGTTACCTTGTTACGACTT (Ramatla et al., 2024a; Mlangeni et al., 2024). PCR reaction consisted of a total reaction of 25 μL consisting of 12.5 μL OneTaq^®^ Quick-Load 2X Master Mix with Standard Buffer (0.4 mM dATP, 0.4 mM dCTP, 0.4 mM dGTP, 0.4 mMdTTP, 4 mM MgCl2, and loading buffer) (New England BioLabs), 8.5 μL of nuclease-free water, 2 μL of the template DNA, and 1 μL of each oligonucleotide primer. The following PCR conditions were used; 95°C for 5 min, 30 cycles of 95°C for 5 min, 55°C for 30 s, 72°C for 1 min and 72°C for 5 min followed by an infinite hold at 4°C. The amplified PCR products were electrophoresed as described above. PCR amplicons were sequenced with the BigDye Terminator sequencing kit v3.1 (Applied Biosystems) on the SeqStudio genetic analyzer at the UESM sequencing facility of North-West University.

### 2.9 Detection of virulence genes from *Pseudomonas aeruginosa*

Six *P. aeruginosa* virulence genes, namely, exoenzyme S (*exoS*), phospholipase N (*plcN*), phospholipase H (*plcH*), exotoxin A (*toxA*), elastas B (*lasB*), and alginate D (*algD*) were screened by PCR from *P. aeruginosa* DNA (Faraji et al., 2016; Ramatla et al., 2024a,b). PCR reaction consisted of a total reaction of 25 μL consisting of 12.5 μL OneTaq^®^ Quick-Load 2X Master Mix with Standard Buffer (0.4 mM dATP, 0.4 mM dCTP, 0.4 mM dGTP, 0.4 mMdTTP, 4 mM MgCl2, and loading buffer) (New England BioLabs), 8.5 μL of nuclease-free water, 2 μL of the template DNA, and 1 μL of each oligonucleotide primer. PCR conditions are listed in Supplementary Table S1. The amplified PCR products were electrophoresed as described above.

### 2.10 Whole genome sequencing and bioinformatics analysis

The MGIEasy FS DNA Prep Kit (BGI, China) was used to create sequence libraries of the two *P. aeruginosa* strains by the manufacturer’s instructions. The strains, P311 and P37, were selected from *Hemipyrellia* spp., flies in residential dumping and livestock kraals sites, respectively. Selection was also based on the isolates’ proven resistance to more than two classes of antibiotics. Using a paired-end 150 nt approach, the produced libraries were sequenced using the BGI MGISEQ-2000 platform (BGI Shenzhen, China). FastQC version 0:10.1 (Andrews, 2017) was used to evaluate the quality of the sequenced reads. The MEGAHIT tool v.1.1.2 (Li et al., 2015) was used to *de novo* assemble the sequence paired-end trimmed reads. For the assembly, kmer sizes 21, 33, 55, 77, 99, and 127 were employed, and the minimum contig length was set at 500 bp. The KBase app (Arkin et al., 2018) was used with CheckM v1.1.6 (Parks et al., 2015) to evaluate individual assembled genomes’ quality and contamination percentage. Quast v 2.3 (Gurevich et al., 2013) assessed the draft genome assemblies. The Pub-MLST^1^ was used to identify the isolates (Jolley et al., 2018). We utilized kSNP within the IPGA tool, employing a k-mer length of 21, a minimum coverage of 10 reads, an error rate threshold of 0.01, genome coverage of at least 95%, and a SNP quality score threshold of 30 to ensure high-confidence SNP identification and accurate phylogenetic analysis as previously described (Magome et al., 2024). Moreover, only genomes with above 90% completeness and <5% contaminants were included in this analysis. We included 10 genomes of *P. aeruginosa* from South Africa isolated from the environment (*n* = 5), currently available, and humans (*n* = 5), including the reference strain PAO1. ABRicate pipeline was employed to screen for antibiotic resistance and virulence genes using the required databases previously described by Fono-Tamo et al. (2023). Briefly, the antibiotic resistance determinants were identified in each assembled genome using the ResFinder [−db ResFinder] (Feldgarden et al., 2019) with the minimum identity and coverage thresholds of 90 (−minid 90) and 70% (−mincov 70), respectively. Virulence factors in the sequenced genomes were mined using the Virulence Factor Database [−db vfdb] (Chen et al., 2005; Liu et al., 2019), using minimum identity and coverage thresholds of 90 (−minid 90) and 70% (−mincov 70), respectively.

### 2.11 Antimicrobial resistance test on *Pseudomonas aeruginosa* isolates

The disk diffusion assay (DDA), also known as the Kirby-Bauer method, was used to determine the antimicrobial profiles of *P. aeruginosa* isolates. Antimicrobial susceptibility was done for the following antibiotics: Aminoglycosides [Neomycin (N), Gentamicin (CN)], β-Lactam [Amoxicillin (AML)], nitroimidazole [Metronidazole (MTZ)], sulfonamide [Sulphamethoxazole (RL)] and cephalosporin [Cefazolin (KZ)]. A standardized inoculum (100 uL) was spread over the surface of Mueller-Hinton agar (MHA). The zone inhibition of the antibiotics was measured in millimeters (mm) according to the Clinical and Laboratory Standards Institute (CLSI), version 2023 (CLSI, 2023). It was classified as multidrug resistant (MDR) if resistant to at least three antibiotic classes (Ramatla et al., 2024b).

### 2.12 Determination of ESBL-producing isolates

ESBL-producing *P. aeruginosa* isolates were confirmed phenotypically on CHROMagar™ ESBL (CHROMagar, France), and plates were incubated aerobically at 37°C for 24 h. The agar differentiates between ESBL *E. coli* (red colony presentation) and ESBL *P. aeruginosa* isolates (blue-green) (Chenhaka et al., 2023). The isolates were chosen because they could produce the ESBL enzymes. The reference strains used in this study were non-pathogenic *E. coli* ATCC 10536 and ESBL-producing *Pseudomonas aeruginosa* ATCC 27853 (Microbiologics, United States).

### 2.13 Detection of antibiotic resistance genes in *Pseudomonas aeruginosa* isolates

The presence or absence of antibiotic-resistance genes was screened from *P. aeruginosa* isolates (Diab et al., 2018; Chow et al., 2001; Rahmani et al., 2013; Ramatla et al., 2024a). PCR reaction consisted of a total reaction of 25 μL consisting of 12.5 μL OneTaq^®^ Quick-Load 2X Master Mix with Standard Buffer (0.4 mM dATP, 0.4 mM dCTP, 0.4 mM dGTP, 0.4 mMdTTP, 4 mM MgCl2, and loading buffer) (New England BioLabs), 8.5 μL of nuclease-free water, 2 μL of the template DNA, and 1 μL of each oligonucleotide primer. PCR conditions and primer sequences are shown in Supplementary Table S2. PCR amplicons were electrophoresed as described above.

## 3 Results

### 3.1 Morpho-species identification of the Diptera flies

A total of 1,422 and 2,793 flies were collected from the illegal residential dumping sites of Ikageng and the livestock kraals of Matlwang village, respectively. Captured Diptera flies, which were identified by microscopy, were categorized based on phenotypic traits such as body color, shape, and size. *Hemipyrellia* spp. (31%), *Tabanus* spp. (24%), and *Chrysomya* spp. (12%) were found in livestock kraals. The illegal residential dumping site revealed the presence of *Hemipyrellia* spp. (88%), *Synthesiomyia* spp. (2%), *Sarcophagidae* spp. (7%), and *Chrysomya* spp. (2%).

### 3.2 Molecular identification of the Diptera flies

An 1,888 bp region of the *CO1* gene was used for genetic identification of the Diptera flies collected from Matlwang and Ikageng in Potchefstroom. BLAST nucleotide analysis identified the sequenced flies belonging to different genera species, supported by percentage identities between query cover and percentage identity with an *E*-value of 0.0. *Hemipyrellia* spp., *Chrysomya* spp., *Synthesiomyia* spp., and *Sarcophagidae* spp. are the four genera and species identified from the residential dumping site (Supplementary Figure S1). Two species belonging to the *Hemipyrellia* genus were found at the dumping site, identified as *H. pulchra* and *H. fernandica. Chrysomya megacephala* was also present at the dumping site, which clustered with *C. megacephala* isolates (MK075818.1 and MK075815.1), which were collected from Shandong in China. Given their close kinship, *Chrysomya* spp. and *Hemipyrellia* spp. belong in the same family as the Calliphoridae. *Synthesiomyia nudiseta* was one of the other taxa determined in this dumping site. The Sarcophagidae comprises *S. tibalias* and *Sarcophagidae* spp. in a different clade (Supplementary Figure S1). Three genera of species were identified in the livestock kraals of Matlwang as *Hemipyrellia* spp., *Chrysomya* spp., and *Tabanus* spp. (Supplementary Figure S2). The *Hemipyrellia* spp., specifically *H. fernandica* and *H. ligurriens*, were shown to belong to the same clade as the *Chrysomya* (*Chrysomya marginals*) in the Matlwang grouping. *Tabanus* spp. formed their own sub-clade, which was identified in this study from the livestock kraals. These were categorized as belonging to the relative minor sub-clades of the species, *T. trivittatus voucher*, *T. birmanicus*, and *T. par*.

### 3.3 Isolation and identification of *Pseudomonas* species using microbiological tests

In total, 59 *Pseudomonas* spp. were isolated and identified from 96 fly samples from residential dumping sites and livestock kraals. *Pseudomonas* spp. were isolated from Diptera flies, namely, *Hemipyrellia* spp., *Synthesiomya* spp., *Chrysomya* spp., and *Sarchophagidae* spp., collected from residential dumping sites. Furthermore, *Pseudomonas* spp. were isolated from Diptera flies, namely *Hemipyrellia* spp., *Chrysomya* spp., and *Tabanus* spp. and collected from the livestock kraals.

### 3.4 Molecular identification of *Pseudomonas aeruginosa* species

A total of 59 *Pseudomonas* spp. isolates from different fly species in the dumping site and livestock kraal were employed for *P. aeruginosa* identification. The species-specific *gyraseB* gene PCR identified 23 and 36 isolates as *P. aeruginosa* isolated from dumping and livestock kraals, respectively. The *16S rRNA* gene PCR and sequencing confirmed these isolates as *P. aeruginosa*. The GenBank accession numbers were assigned to some of the represented strains (*n* = 5) as follows: sequence 1: OR122642; sequence 2: OR122643; sequence 3: OR122644; sequence 4: OR122645; sequence 5: OR122646.

### 3.5 Detection of virulent genes in *Pseudomonas aeruginosa* isolates by PCR

**Figure 2:**
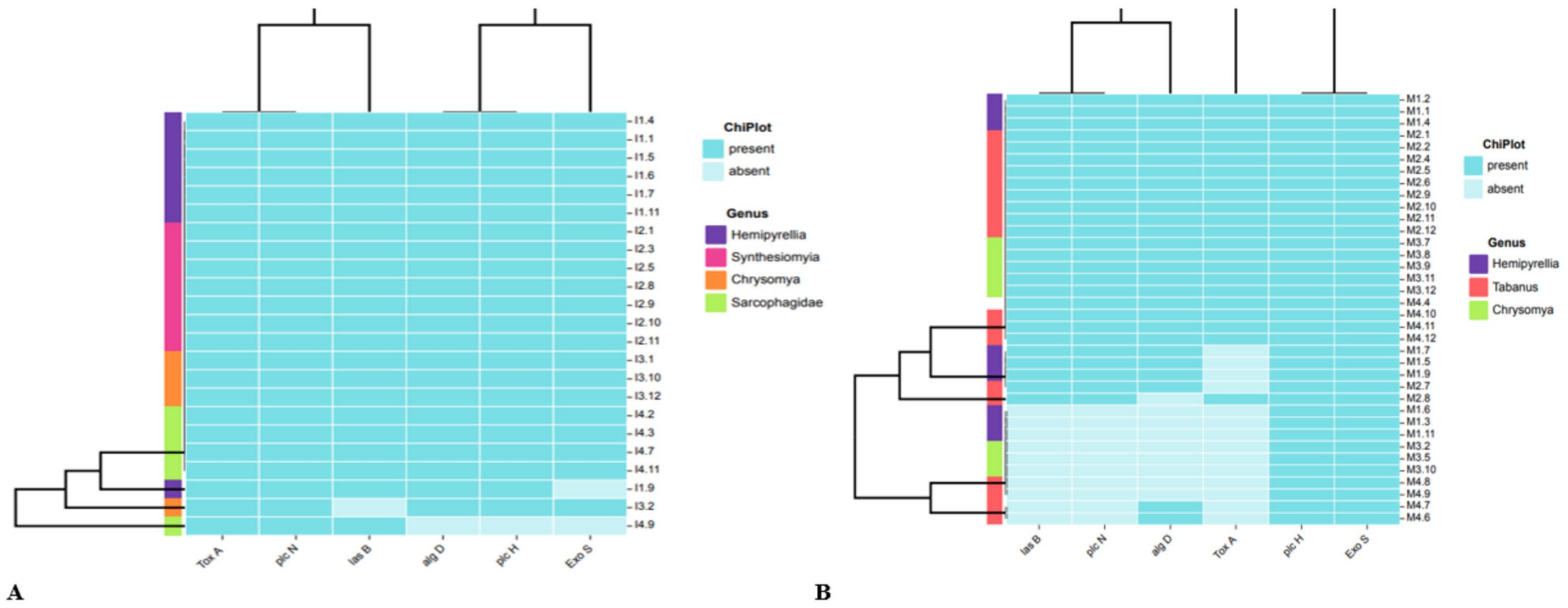
Phylogenetic clustering of the virulence genes that are present or absence in *P. aeruginosa* isolates in relation to fly-genera collected from **(A)** residential dumping site and **(B)** livestock kraals.

About 23 isolates from a residential dumping site from different fly genera, namely, *Hemipyrellia* spp. (*n* = 7), *Synthesiomyia* spp. (*n* = 7), *Chrysomya* spp. (*n* = 4) and *Sarcophagidae* spp. (*n* = 5), were tested for the presence and absence of these virulent genes. The exotoxin (*toxA*) and phospholipase (*plcN*) genes were detected in all (*n* = 23) *P. aeruginosa* isolates collected from residential dumping sites (Figure 2A). The *elastase* (*lasB*) gene was detected in all the *P. aeruginosa* isolates from *Hemipyrellia* spp., *Sarcophagidae* spp., and *Synthesiomyia* spp. The *lasB* gene was detected in (*n* = 3; 75%) of *P. aeruginosa* isolated from *Chrysomya* spp. The hemolytic phospholipase C (*plcH*) and the GDP-mannose 6-dehydrogenase (*algD*) gene were detected in all (100%) of the *P. aeruginosa* isolates from *Chrysomya* spp., *Hemipyrellia* spp. and *Synthesiomyia* spp. The *plcH* gene was present in (*n* = 4; 80%) of the isolates from *Sarcophagidae* spp. The *exoenzyme S* (*exoS*) gene was detected in all the isolates from fly-genera *Synthesiomyia* spp. and *Chrysomya* spp. (100%), *Hemipyrellia* spp. (*n* = 6; 86%), and present in (*n* = 4; 80%) of the *Sarcophagidae* spp.

The livestock kraals identified 36 *P. aeruginosa* isolated from different fly genera, *Hemipyrellia* spp. (*n* = 9), *Tabanus* spp. (*n* = 17), and *Chrysomya* spp. (*n* = 8) and were profiled for the virulence genes (Figure 2B). The *plcH* and *exoS* genes were present in isolates investigated. The *lasB* gene was detected in *Hemipyrellia* spp. (*n* = 6; 66.7%), *Tabanus* spp. (*n* = 13; 76.47%), and *Chrysomya* spp. (*n* = 5; 62.5%), respectively. The *algD* gene was present in *Hemipyrellia* spp. (*n* = 6; 66.7%), *Tabanus* spp. (*n* = 14; 84.3%), and *Chrysomya* spp. (*n* = 5; 62.5%), respectively. The *toxA* gene was detected in 3 (33.33%) isolates from *Hemipyrellia* spp., (*n* = 12; 70.6%) in *Tabanus* spp., and (*n* = 5; 62.5%) in *Chrysomya* spp.

### 3.6 Antimicrobial susceptibility testing of *Pseudomonas aeruginosa* and determination of ESBL-producing isolates

**Figure 3:**
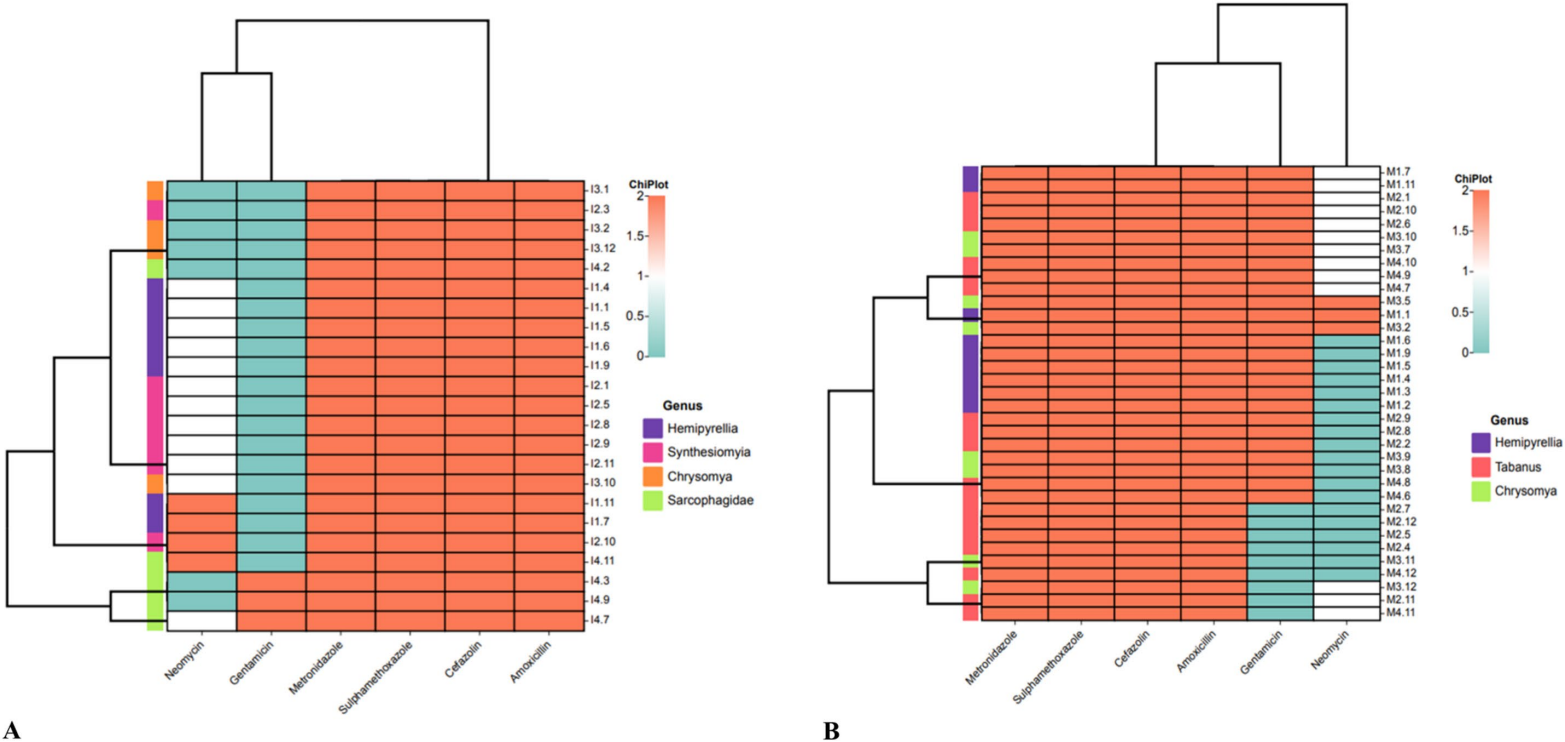
Heatmap analysis of the antibiotic susceptibility using the Kirby-Bauer method for *P. aeruginosa* isolates from flies collected at **(A)** residential dumping site and **(B)** livestock kraals.

An antimicrobial susceptibility test was performed to profile the 59 *P. aeruginosa* isolates isolated from residential dumping (Figure 3A) and livestock kraal sites (Figure 3B). All *P. aeruginosa* isolates investigated in this study were resistant to metronidazole, sulphamethoxazole, cefazolin, and amoxicillin. Thirteen percent of *P. aeruginosa* isolates from residential dumping sites were resistant to gentamicin, while 38.9% were resistant to neomycin. Meanwhile, 30.4 and 52.2% of isolates were classified as susceptible and intermediate to neomycin, respectively. Furthermore, 38.9% of *P. aeruginosa* isolated from livestock kraals were resistant to gentamicin. The extended-spectrum beta-lactamases (ESBL) were determined among all the confirmed *P. aeruginosa* isolates (*n* = 59). All the investigated *P. aeruginosa* isolates were considered to be ESBL-producing.

### 3.7 Antibiotic resistance genes in *Pseudomonas aeruginosa* isolates detected by PCR

Four different antibiotic resistance genes (ARGs) were investigated in the *P. aeruginosa* isolates collected from residential dumping site (*n* = 23) and livestock kraal sites (*n* = 36). In this study, the detected ARGs included neomycin *(aph (2″)-Ib)*, gentamicin *(acc(3)-IV)*, metronidazole *(rdxA)*, sulphamethoxazole *(sulI)* and amoxicillin *(pbp1A)*. All *P. aeruginosa* isolates collected from residential dumping sites tested positive for the aminoglycoside *acc(3)-IV* gene that confers resistance to gentamicin. The PCR assay revealed the presence of the *rdxA* (43.5%) and *sulI* (86.9%) genes. The absence of the *aph(2″)-Ib* gene in *P. aeruginosa* isolates indicates their susceptibility to neomycin. The lack of the *pbp1A* gene is inconsistent with the DDA, whereby most of the isolates appeared to be resistant to amoxicillin.

Three ARGs were detected in *P. aeruginosa* isolates from livestock kraal, including *acc(3)-IV* (80.6%) and *sulI* (88.9%), genes that confer resistance to gentamicin and sulphamethoxazole, respectively. The *pbp1A* and *aph (2″)-Ib* genes that confer resistance to amoxicillin and neomycin, respectively, were absent in all the *P. aeruginosa* isolates. The absence of *pbp1A* gene is inconsistent with the disk diffusion assay, whereby all the isolates were resistant to amoxicillin. Four different β-lactam genes were examined among *P. aeruginosa* isolates from residential dumping (*n* = 23) and livestock kraal sites (*n* = 36) flies. The 4 β-lactam genes included *bla*_OXA_, *bla*_SHV_, *bla*_TEM_, and *bla*_CTX-M_. Only one *P. aeruginosa* isolate from the fly genus, *Hemipyrellia* spp. collected from livestock kraal consisted of the *bla*_CTX-M_ gene.

### 3.8 Genome assembly of the *Pseudomonas aeruginosa* isolates

With an average of 150 bp paired-end reads, a total of 2,865,578 sequence reads for strain P311 and 2,996,628 for strain P7 were generated on the MGI sequencer. After trimming the sequence reads, 2,841,220 and 2,953,424 reads were determined from the strains P311 and P7, respectively. The genome features show that strain P37 is approximately 6.45 Mb, while strain P311 is ~6.37 Mb, which are both higher than the reference strain PAO1 which is 6.26 Mb. Strain P37 has a higher GC content, impacting the number of coding sequences determined. The G + C content of the sequenced strains and reference strain PAO1 is ~66%. Genome sequences of the two strains P37 and P311 isolated from *Hemipyrellia* spp. have been deposited in GenBank under the accession numbers JBDJPE000000000 *P. aeruginosa* P37 and JBDJPD000000000 *P. aeruginosa* P311. Genome assemblies are shown in Supplementary Table S3. Both the sequenced strains belong to the sequence type ST3808. The two strains group closely with *P. aeruginosa* strain WW isolated from South Africa, North West from water source.

### 3.9 Detection of virulence genes in *Pseudomonas aeruginosa* by whole genome sequencing

**Figure 4:**
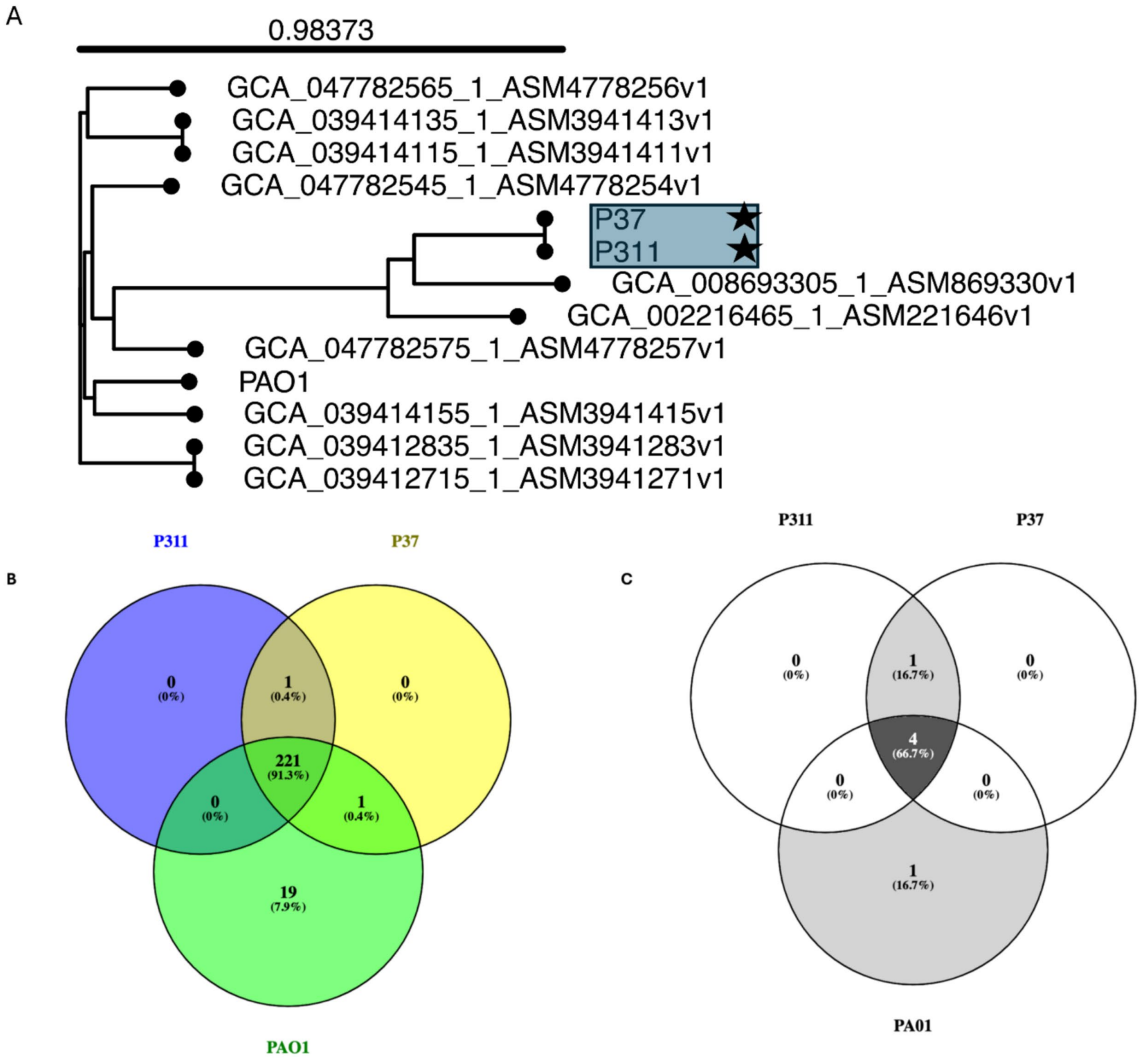
Whole genome-based phylogenetic tree using single nucleotide polymorphism inferring the evolutionary relationships among the sequenced *Pseudomonas aeruginosa* strains with antibiotic-resistant and virulence determinates. **(A)** Genome Blast Distance Phylogenies (GBDP) identified by TYGS between the sequenced *P. aeruginosa strains (indicated by user strain) and* related genomes. **(B)** Virulence genes and **(C)** antibiotic resistance genes that are shared among the sequenced strain and reference strain PAO1.

The use of whole genome sequencing on the selected *P. aeruginosa* strains isolated from *Hemipyrellia* spp. was confirmed as *P. aeruginosa* (Figure 4A). The strains clustered with *P. aeruginosa* strain ww isolated from water in the same Province. Whole genome sequencing identified 221 virulence genes in the sequenced *P. aeruginosa* P311 and P37 strains and the reference strain PAO1 (Figure 4B). Shared genes included flagellar motor proteins (MotC and MotD), flagellar biosynthetic proteins, type III secretion system proteins, phenazine biosynthesis proteins, paerucumarin biosynthesis proteins, pyoverdine biosynthesis proteins, general secretion pathway proteins, etc. In this study, only the type III secretion system effector ExoU phospholipase A2 activity was common in the sequenced strains, i.e., P311 and P37. About 19 virulence genes were unique to the reference strain PAO1. These include genes such as the (wzy) O-antigen chain length regulator, the type 4 fimbrial precursors PilA and PilB, the pyoverdine biosynthesis protein PvdJ- PvdD- PvdI complex, and the type VI secretion system substrate VgrG1b.

### 3.10 Antibiotic resistance genes detected by whole genome sequencing

The use of whole genome sequencing showed that the two sequenced *P. aeruginosa* strains, i.e., P37 and P311 are resistant to fosfomycin (*fosA_4*), chloramphenicol (*catB_1*), ampicillin (bla_OXA-50_1_), beta-lactamase (*bla_PAO_*), and aminoglycoside (*aph(3′)-IIb_2*) (Figure 4C; Supplementary Table S4). Moreover, the identified ARG profiles on the draft genomes of the sequenced *P. aeruginosa* strains were also found on the reference genome of *P. aeruginosa* strain PAO1 (AE004091.2). Differences were observed in the sequenced strains P311 and P37, consisting of the shared beta-lactamase *blaPAO_4*, while the reference PAO has the *blaPAO_2* gene.

## 4 Discussion

This study investigated the antimicrobial resistance and virulence profiles of *P. aeruginosa* associated with the gut of Diptera flies collected from livestock kraals and residential dumping sites. *Pseudomonas aeruginosa* is an important opportunistic pathogen and a key factor in both nosocomial and community-acquired infections. Although it is frequently examined in clinical settings, its environmental reservoirs remain inadequately explored, especially in flies from anthropogenically impacted areas. This study addresses that gap, contributing to a One Health perspective on pathogen transmission dynamics at the intersection of humans, animals, and the environment. The study sheds light on how poor waste management in peri-urban settings contributes to the environmental persistence and amplification of *P. aeruginosa*, positioning these environments as critical, yet often neglected, hotspots in the antimicrobial resistance (AMR) cycle. This study shows that Diptera flies in residential and livestock-associated environments are carriers of multidrug-resistant *Pseudomonas aeruginosa*, harboring a range of antibiotic-resistance genes.

In this study, a significantly higher number of Diptera flies were associated with residential dumping sites as opposed to livestock kraals. This likely reflects the nutrient-rich and unhygienic conditions of dumping sites, which offer abundant resources for breeding (Goulson et al., 1999). In contrast, only a small number of Diptera species were linked to livestock, which suggests livestock kraals as a harsher habitat with fewer provisions and breeding space, where Diptera flies that are suited for such conditions might flourish, especially those that feed on feces and blood (Shety et al., 2022). The high density of flies in peri-urban dumping areas indicates a risk of pathogen transfer between human communities and nearby livestock operations, emphasizing the necessity of effective waste management for disease control.

Among the Diptera, *Hemipyrellia* spp. were found in substantial quantities in livestock kraal (31%) and the illegal residential dumping site (88%). They are known to proliferate in environments that include waste materials, dead animals, as well as animal and human excrement (Hore et al., 2017). Twenty-four percent of the *Tabanus* spp., also known as horseflies, were found exclusively in the livestock kraals. Female *Tabanus* spp. normally require a blood meal to reproduce, making them potential carriers of pathogenic bacteria (Snyman et al., 2020). In warm, humid regions of South Africa, such as the tropical climate of KwaZulu-Natal province, *Tabanus* spp. is highly prevalent (Esterhuizen, 2006). Seven percent of the *Sarcophagidae* spp. that belong to the family known as flesh flies were found in this study. Additionally, reports of *Sarcophagidae* spp. have been made in Cusco, Peru’s residential areas (Ly et al., 2018). Since *Sarcophagidae* spp. are ovoviviparous, they opportunistically deposit hatching or hatched maggots rather than eggs (Ren et al., 2018). The dumping site is an ideal environment for them to breed, given that there is an abundance of decaying and unsanitary materials to offer the maggots the highest chance of surviving (Ren et al., 2018). They typically deposit on carrion, decaying material, and feces. The detection of *P. aeruginosa* in environmentally abundant fly species such as *Musca domestica* and *Hemipyrellia* spp. suggests that these insects may play an active role in the environmental circulation of clinically significant resistance genes.

*Pseudomonas aeruginosa* is a known opportunistic pathogen with different virulence factors that enable it to reside in various host niches. These bacteria are a major global source of nosocomial and community-acquired illnesses. The virulence genes exotoxin A *(toxA)* and phospholipase N (*plcN*) were detected in all investigated isolates. The *toxA* gene is responsible for the regulation of exotoxin A synthesis and is known to cause tissue damage effectively and decrease the phagocytic activity of leukocytes in infected patients (Dong et al., 2015). Multidrug-resistant (MDR) *P. aeruginosa* strains isolated from patients have a substantially greater frequency of the *toxA* gene (76.6% of MDR strains) (Khosravi et al., 2016). However, there are no reports of the prevalence of *toxA* gene in Diptera flies. The *plcN* gene encodes for the phospholipase C enzyme, which disrupts host cell membranes and contributes to tissue damage (Terada et al., 1999). Another type of phospholipase is the *plcH* gene, which is responsible for hydrolysis phospholipids. This gene was detected in 99% of the isolates investigated in this study. The *P. aeruginosa* isolates in this study lacked the *plcH* gene and were augmented with other virulent genes that were absent, including alginate lyase (*algD*) and exoenzyme S (*exoS*). The *P. aeruginosa* is protected from the host defense mechanism by the *algD* gene during biofilm formation. This is achieved by the reduction of phagocytosis as well as the inhibition of the activation of complement proteins (Edward et al., 2023). While *exoS* impairs phagocytosis in the lungs, exacerbating infection (Faraji et al., 2016).

The *lasB* gene, encoding elastase that disrupts immune function, was found in 99% of *P. aeruginosa* isolated from Diptera flies collected from residential dumping sites. The virulence factor *lasB* in *P. aeruginosa* is responsible for proteolytic actions ranging from tissue damage to compromising the host immune system (Cathcart et al., 2011). However, this gene was absent in 16.6% of isolates from *Hemipyrellia* spp., *Tabanus* spp. (36%), and *Chrysomya* spp. (13.8%) in livestock kraals. The latter-mentioned strains, which lack other virulent genes such as *toxA*, *lasB*, and *plcN*, suggest that these *P. aeruginosa* can be classified as avirulent strains. The avirulent *P. aeruginosa* strains (70.3%) have been identified in clinical host material (Luzar and Montie, 1985). Nevertheless, these strains in the current study cannot be neglected as they showed multiple resistances to various antibiotics. This variation in virulence gene profiles suggests the circulation of both virulent and avirulent *P. aeruginosa* strains in environmental fly populations, mirroring clinical findings (Luzar and Montie, 1985) and underscoring the complex ecology of this pathogen.

*Pseudomonas aeruginosa* sensitivity to neomycin can vary based on a number of variables, such as the strain of the bacterium, its genetic composition, and any acquired resistance mechanisms it may have (Pang et al., 2019). Neomycin is effective against a wide range of bacterial strains, including certain isolates of *P. aeruginosa* (Uemura et al., 2017). This was evident as most isolates (88.14%) examined in this study were either intermediate or susceptible to neomycin. Furthermore, *P. aeruginosa* can withstand elevated neomycin concentrations and demonstrates enduring adaptive resistance, which results in cross-resistance to additional aminoglycoside antibiotics (Uemura et al., 2017). This should further be exploited using the minimum inhibition concentration assay, particularly as *P. aeruginosa* isolated from Diptera flies is not well-investigated globally. Based on DDA, all isolates were resistant to amoxicillin, supported by the *bla*_OXA-50_ gene, which was noticeable in the two genome-sequenced strains. The *P. aeruginosa* isolated from canine clinical cases found in South Africa also showed resistance against amoxycillin-clavulanic acid (99%) (Eliasi et al., 2020). Moreover, all isolates characterized in this study were considered ESBL-producers. However, these isolates did not detect the amoxicillin (*Pbp1A*) gene. In *P. aeruginosa*, the *PbplA* gene encodes a penicillin-binding protein. Enzymes called penicillin-binding proteins (PBPs) are part of the bacterial cell wall production process (Handfield et al., 1997). Beta-lactam antibiotics, such as cephalosporins and penicillins are directed toward them. One of the PBPs involved in cell wall formation in *P. aeruginosa* is *PbplA*, which is inhibited by beta-lactam antibiotics, preventing bacterial growth and causing cell death. A discrepancy between phenotypic and resistance to amoxicillin and the molecular detection of the *Pbp1A* gene by PCR. While PCR failed to amplify *Pbp1A* in several phenotypically resistant isolates, whole genome sequencing (WGS) of the two sequenced isolates confirmed the presence of *Pbp1A* and *Pbp1B*, as well as the associated lipoprotein activator *LpoP*, which is essential for the proper function of PBP1A. This inconsistency suggests that the PCR-based approach may have been limited by suboptimal primer design, possibly due to genetic variability in primer binding regions or sequence divergence in local *P. aeruginosa* populations. These findings highlight the importance of validating molecular detection methods against WGS and point to redesigning or optimizing primers to ensure accurate gene detection, mainly when correlating genotypic data with antibiotic resistance phenotypes. This discrepancy emphasizes the need to align molecular diagnostics with genomic evidence, particularly for environmental surveillance applications. The *bla*_CTX-M_ gene was present among *P. aeruginosa* isolates (4.35%) harbored by flies from residential dumping sites and livestock kraals. The *bla*_CTX-M_ is the least frequently isolated ESBL gene in *P. aeruginosa* strains among antibiotic-resistant strains (Shacheraghi et al., 2010). The lower prevalence of *bla*_CTX-M_ gene (4.35%) in this study differs from that reported on isolates from patients in Mthatha City, Eastern Cape Province in South Africa, whereby 31.7% of the isolates tested positive (Hosu et al., 2021).

The *acc(3)-IV* gene is a type of aminoglycoside acetyltransferase encoded by the *acc(3)-IV* gene in bacteria, including *P. aeruginosa*. This enzyme acetylates aminoglycoside antibiotics, modifying them and decreasing their potency. In this study, about 88% of the isolates from the residential dumping site and the livestock kraal tested positive for this gene. Based on DDA, the isolated *P. aeruginosa* isolates (38.9%) from livestock kraals were also resistant to gentamicin, while 87% of the isolates from dumping sites were susceptible. Resistance to gentamicin has also been reported from *P. aeruginosa* isolated from dairy cattle, milk, the environment, and workers’ hands in Egypt (Badawy et al., 2023). In Iran, *P. aeruginosa* isolates from *M. domestica* flies showed a 49.1% resistance rate (Hemmatinezhad et al., 2015). Gentamicin is a routinely used antibiotic for treating *P. aeruginosa* infections; the existence of gentamicin-resistant *P. aeruginosa* strains is alarming, as resistance to this antibiotic can restrict treatment options and make managing the bacterium’s infections more difficult.

The *sul1* gene, which is linked to sulfamethoxazole resistance, was detected in 87.72% of the *P. aeruginosa* isolates in the current study (Rahmani et al., 2013). Based on DDA, all isolates were confirmed to be resistant to sulphamethoxazole. The leading causes of *P. aeruginosa* resistance to sulfamethoxazole are mutations in topoisomerases, decreased expression of outer membrane proteins, and the synthesis of beta-lactamases and enzymes that alter aminoglycosides (Bonomo and Szabo, 2006). Nevertheless, none of the *sul1* gene was detected in the whole genome sequenced strains of *P. aeruginosa* in this study, suggesting that isolates do not necessarily express gene mutations similarly or simultaneously. Whole genome sequencing of two sequenced *P. aeruginosa* isolates revealed that both belong to sequence type ST3808. This sequence type has not been widely reported in fly-associated *P. aeruginosa* and may represent an emerging lineage in non-clinical environments. Notably, phylogenetic analysis based on single nucleotide polymorphisms showed that the two ST3808 strains clustered closely with *P. aeruginosa* strain WW, a 2017 genome that was isolated from a water source in the North West province of South Africa.

This study significantly contributes to the One Health discourse by highlighting Diptera flies as often-overlooked vectors of AMR *P. aeruginosa*. Detecting clinically relevant resistance genes and virulence factors in flies from livestock and residential environments demonstrates the pathogen’s ability to cross ecological boundaries, reinforcing its zoonotic and environmental threat (Lister et al., 2009; Santajit and Indrawattana, 2016). Importantly, the study emphasizes several key implications for One Health. It establishes the zoonotic potential of Diptera flies, which can act as vectors for transmitting resistant *P. aeruginosa* among livestock, environmental sources, and human communities. Additionally, it points to the role of environmental reservoirs, particularly in peri-urban areas where inadequate waste management promotes the proliferation and persistence of pathogens.

The limitations of this study stem from excluding fly species that were not shared between the two sites in order to maintain comparability, we may have overlooked significant species-specific variations in pathogen carriage, thereby narrowing the ecological scope of our findings. Furthermore, excluding environmental and host samples, whether from livestock or humans, restricts our understanding of broader transmission dynamics and potential reservoirs within the One Health context, which merits further exploration. Moreover, the discrepancies noted between phenotypic antibiotic resistance profiles and genotypic findings, such as the resistance to amoxicillin in the absence of the *pbp1A* gene, underscore the complexity of antimicrobial resistance mechanisms that targeted PCR assays may not comprehensively capture. While whole genome sequencing was employed, only two isolates were analyzed, potentially limiting the representation of the genomic diversity of *P. aeruginosa* in these environments; subsequent work will involve sequencing additional isolates.

## 5 Conclusion

This study demonstrates that synanthropic Diptera fly species collected from illegal residential dumping sites and livestock kraals harbor multidrug-resistant *P. aeruginosa*, highlighting their potential role as vectors in disseminating clinically significant pathogens. Phenotypic analysis confirmed resistance to several antibiotics, notably amoxicillin, while molecular screening identified resistance genes such as *bla_TEM*, *bla_SHV*, *bla_CTX-M*, and efflux pump-related genes (*mexA*, *oprM*). This bacterium is well-known for being resilient and adaptable, especially in situations containing a significant number of organic materials. This bacterium is essential to “One Health” due to its multidrug resistance character and zoonotic nature. These findings suggest that flies could play a significant role in the environmental spread of antibiotic resistance, emphasizing the need for integrated “One Health” surveillance approaches that consider the interface of human, animal, and ecological health.

## Data Availability

The datasets presented in this study can be found in online repositories. The names of the repository/repositories and accession number(s) can be found at: https://www.ncbi.nlm.nih.gov/, JBDJPE000000000, JBDJPD000000000, OR122642–OR122646, MK075818.1h, and MK075815.1.

## Footnotes

^1^ https://pubmlst.org/species-id
