# Supplementary material for: Uncovering antibiotic resistance: extended-spectrum beta-lactamase-producing Pseudomonas aeruginosa from dipteran flies in residential dumping and livestock environments
Source: Front Microbiol. 2025 Jun 4;16:1586811. doi: 10.3389/fmicb.2025.1586811 (PMC12174077; doi:10.3389/fmicb.2025.1586811)
Supplement: Supplementary file 1 [file Data_Sheet_1.DOCX]

**Uncovering antibiotic resistance: Extended-spectrum beta-lactamase-producing *Pseudomonas aeruginosa* from Dipteran flies in residential dumping and livestock environments**

Lara de Wet^1^, Itumeleng Matle^2^, Oriel Thekisoe ^1^, Kgaugelo E. Lekota^1^, Tsepo Ramatla^1,3^

^1^ Unit for Environmental Sciences and Management, North-West University, Potchefstroom, 2531, South Africa.

^2^ Bacteriology Division, Onderstepoort Veterinary Research, Agricultural Research Council, 100 Old Soutpan Road, Onderstepoort 0110, South Africa

^3^ Centre for Applied Food Safety and Biotechnology, Department of Life Sciences, Central University of Technology, 1 Park Road, Bloemfontein, 9300, South Africa.

Corresponding Author*-email: [ra21205450@gmail.com](mailto:ra21205450@gmail.com)

**Supplementary Table S1:** *Pseudomonas aeruginosa* virulence genes primers, and PCR conditions used in this study.

| **Target gene** | **Primer name** | **Sequences** | **PCR conditions** | **Base pairs** | **References** |
| --- | --- | --- | --- | --- | --- |
| *algD* | algD-F:  algD-R: | CGTCTGCCGCGAGATCGGCT | 94°C - 1 min (30x)  60°C - 1.5 min  72°C - 1 min | 313 bp | Faraji *et al*., 2016 |
|  |  | GACCTCGACGGTCTTGCGGA |  |  |  |
| *lasB* | lasB-F:  lasB-R: | GGAATGAACGAAGCGTTCTCCGAC | 94°C - 1 min (30x)  60°C - 1.5 min  72°C - 1 min | 284 bp | Faraji *et al*., 2016 |
|  |  | TTGGCGTCGACGAACACCTCG |  |  |  |
| *toxA* | toxA-F:  toxA-R: | CTGCGCGGGTCTATGTGCC | 94°C - 1min (30x)  63°C - 1.5 min  72°C - 1 min | 270 bp | Faraji *et al*., 2016 |
|  |  | GATGCTGGACGGGTCGAG |  |  |  |
| *plcH* | plc H-F:  plc H-R: | GCACGTGGTCATCCTGATGC | 94°C - 1 min (30x)  60°C - 1.5 min  72°C - 1 min | 608 bp | Faraji *et al*., 2016 |
|  |  | TCCGTAGGCGTCGACGTAC |  |  |  |
| *plcN* | plc N-F:  plc N-R: | TCCGTTATCGCAACCAGCCCTACG | 94°C - 1 min (30x)  60°C - 1.5 min  72°C - 1 min | 481 bp | Faraji *et al*., 2016 |
|  |  | TCGCTGTCGAGCAGGTCGAAC |  |  |  |
| *exoS* | exo S-F:  exo S-R: | CGTCGTGTTCAAGCAGATGGTGCTG | 94°C - 1 min (30x)  60°C - 1.5 min  72°C - 1 min | 444 bp | Faraji *et al*., 2016 |
|  |  | CCGAACCGCTTCACCAGGC |  |  |  |

**Supplementary Table S2:** Antimicrobial resistant confirmation genes in *Pseudomonas aeruginosa* isolates

| **Antibiotic name** | **Primer name** | **PCR conditions** | **Sequence** | **References** |
| --- | --- | --- | --- | --- |
| Gentamicin | AME 14ACC(3) IV R  AME 13ACC(3) IV F | 95°C 3min  95°C 1min x 35  54°C 1min  72°C 5min  72°C 10min | F: AGTTGACCCAGGGCTGTCGC  R: GTG TGC TGC TGG TCC ACA GC | Bräu *et al.,* 1984 |
| Amoxicillin | Pbp1A F  Pbp1A R | 95°C 3min  95°C 1min x 35  54°C 1min  72°C 5min  72°C 10min | F: GCG ACA ATA AGA GTG GCA  R: TGC GAA CAC CCT TTT AAA T | Diab *et al.,* 2018 |
| Neomycin | aph(2′′)-Ib F  aph(2′′)-Ib R | 94°C 4min  94°C 1min x 30  55°C 2min  72°C 1min  72°C 10min | F: CTTGGACGCTGAGATATATGAGCAC  R: GTTTGTAGCAATTCAGAAACACCCTT | Chow *et al.,* 2001 |
| Sulphamethoxazole | sulI F  sulI R | 95°C 1min 30sec  95°C 30sec x 40  65°C 30sec  72°C 30sec  72°C 7min | F: CGCACCGGAAACATCGCTGCAC  R: TGAAGTTCCGCCGCAAGGCTCG | Rahmani *et al.,* 2013 |
| *OXA* | OXA – F  OXA – R | 94°C 5min  94°C 45s x35  55°C 30sec  72°C 1min  72°C 10min | F: ACACAATACATATCAACTTCGC  R: AGTGTGTTTAGAATGGTGATC | Liu *et al*., 2018 |
| *SHV* | SHV – F  SHV – R | 94°C 5min  94°C 45s x35  55°C 30sec  72°C 1min  72°C 10min | F: CACTCAAGGATGTATTGT G  R: TTAGCGTTGCCAGTGCTCG | Liu *et al*., 2018 |
| *TEM* | TEM-F  TEM-R | 94°C 5min  94°C 45s x35  55°C 30sec  72°C 1min  72°C 10min | F: TTC TTG AAG ACG AAA GGG C  R: ACGCTCAGTGGAACGAAAAC | Liu *et al*., 2018 |
| *CTX-M* | CTX-M-F  CTX-M-R | 94°C 5min  94°C 45s x35  55°C 30sec  72°C 1min  72°C 10min | F: GTTACAATGTGTGAGAAGCAG  R: CCGTTTCCGCTATTACAAAC | Liu *et al*., 2018 |

**Supplementary Table S3:** De novo assembly statistics and genomic features of the sequenced *Pseudomonas aeruginosa* isolates.

| **Strain** | **P37_** | **P311_** | **PAO1*** |
| --- | --- | --- | --- |
| Sequence reads |  |  |  |
| # contigs | 85 | 40 | 1 |
| # contigs (>= 0 bp) | 105 | 40 | 1 |
| # contigs (>= 1000 bp) | 57 | 40 | 1 |
| Largest contig | 1,040,117 | 1,040,133 | 6,264,404 |
| Total length | 6,457,649 | 6,378,458 | 6,264,404 |
| Total length (>= 0 bp) | 6466380 | 6378458 | 6264404 |
| Total length (>= 1000 bp) | 6439511 | 6378458 | 6264404 |
| N50 | 334 498 | 334 512 | 6,264,404 |
| GC (%) | 66.37 | 66.5 | 66.56 |
| Total CDSs | 6,051 | 5,940 | 5,570 |
| Accession # | JBDJPE000000000 | JBDJPD000000000 | AE004091.2 |

* PAO1 was used as a reference.

**Supplementary Table S4**: Antibiotic resistant genes determined on the whole genome sequences of the *Pseudomonas aeruginosa* sequenced in this study.

| **Genome/ Strain** | **Sequence** | **Gene** | **Coverage** | **%Coverage** | **%Identity** | **Product** | **Resistance** |
| --- | --- | --- | --- | --- | --- | --- | --- |
| P311 | k141_108 | *fosA_4* | 1-408/408 | 100.00 | 99.51 | *fosA* | Fosfomycin |
| P311 | k141_108 | *catB7_1* | 1-639/639 | 100.00 | 98.44 | *catB7* | Chloramphenicol |
| P311 | k141_112 | *bla_OXA-50_1_* | 1-789/789 | 100.00 | 99.37 | *blaOXA-50* | Amoxicillin; Ampicillin |
| P311 | k141_134 | *bla_PAO_4_* | 1-1194/1194 | 100.00 | 99.25 | *blaPAO* | Betalactamase |
| P311 | k141_134 | *aph(3')-IIb_2* | 1-807/807 | 100.00 | 99.63 | *aph(3')-IIb* | Aminoglycoside |
| P37 | k141_104 | *bla_OXA-50_1_* | 1-789/789 | 100.00 | 99.37 | *blaOXA-50* | Amoxicillin;Ampicillin |
| P37 | k141_114 | *catB7_1* | 1-639/639 | 100.00 | 98.44 | *catB7* | Chloramphenicol |
| P37 | k141_114 | *fosA_4* | 1-408/408 | 100.00 | 99.51 | *fosA* | Fosfomycin |
| P37 | k141_8 | *blaPAO_4* | 1-1194/1194 | 100.00 | 99.25 | *blaPAO* | Betalactamase |
| P37 | k141_8 | *aph(3')-IIb_2* | 1-807/807 | 100.00 | 99.63 | *aph(3')-IIb* | Aminoglycoside |
| PAO1* | AE004091.2 | *catB7_1* | 1-639/639 | 100.00 | 100.00 | *catB7* | Chloramphenicol |
| PAO1 | AE004091.2 | *fosA_4* | 1-408/408 | 100.00 | 99.51 | *fosA* | Fosfomycin |
| PAO1 | AE004091.2 | *blaPAO_2* | 1-1194/1194 | 100.00 | 100.00 | *blaPAO* | Betalactamase |
| PAO1 | AE004091.2 | *aph(3')-IIb_2* | 1-807/807 | 100.00 | 100.00 | *aph(3')-IIb* | Aminoglycoside |
| PAO1 | AE004091.2 | *bla_OXA-50_1_* | 1-789/789 | 100.00 | 99.87 | *blaOXA-50* | Amoxicillin;Ampicillin |

- Strain PAO1 was used as a reference genome comparative genomics analysis.

**
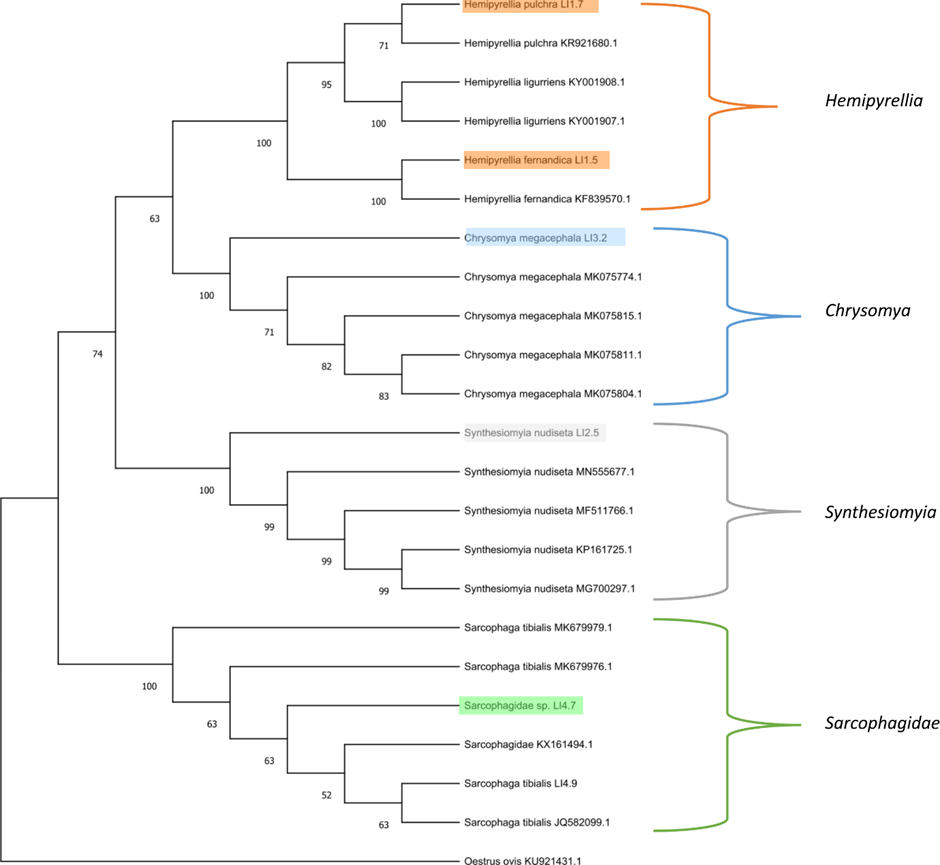
Supplementary Figure S1**: Using the *cytochrome oxidase 1* (*CO1*) gene, a neighbor joined phylogenetic tree of fly species collected from Ikageng residential dumping site is sequenced. At the internodes, bootstrap support values (>63%) are displayed. The study's sequenced samples are highlighted. To root the tree, the Oestrus KU921431.1 was employed as an outgroup.

**
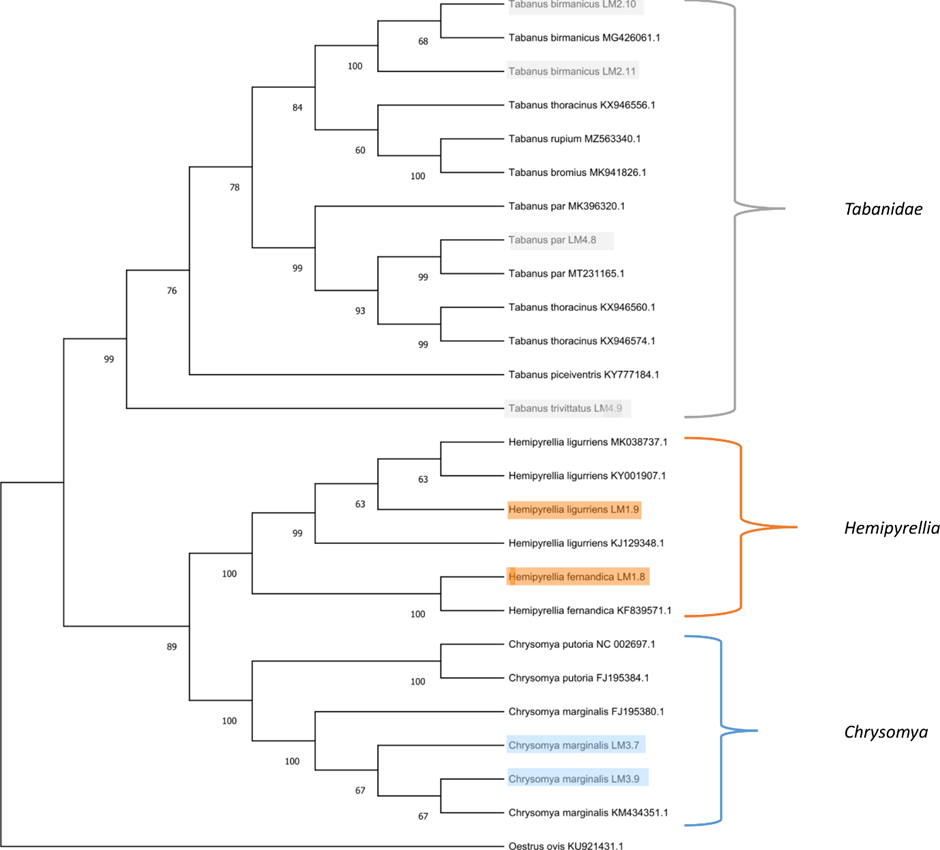
Supplementary Figure S2:** Employing the *cytochrome oxidase 1* (*CO1*) gene, a neighbour joining phylogenetic tree was obtained from the fly genus species collected in Matlwang livestock kraals. At the internodes, bootstrap support values (>49%) are displayed. The study's sequenced samples are highlighted. To root the tree, the Oestrus ovis KU921431.1 was employed as an outgroup.
